# Longitudinal Changes of COVID-19 Symptoms in Social Media: Observational Study

**DOI:** 10.2196/33959

**Published:** 2022-02-16

**Authors:** Sarah Sarabadani, Gaurav Baruah, Yan Fossat, Jouhyun Jeon

**Affiliations:** 1 Applied Sciences Klick Inc Toronto, ON Canada

**Keywords:** COVID-19, symptom, diagnosis, treatment, social media, Reddit, longitudinal, observational, machine learning

## Abstract

**Background:**

In December 2019, the COVID-19 outbreak started in China and rapidly spread around the world. Many studies have been conducted to understand the clinical characteristics of COVID-19, and recently postinfection sequelae of this disease have begun to be investigated. However, there is little consensus on the longitudinal changes of lasting physical or psychological symptoms from prior COVID-19 infection.

**Objective:**

This study aims to investigate and analyze public social media data from Reddit to understand the longitudinal impact of COVID-19 symptoms before and after recovery from COVID-19.

**Methods:**

We collected 22,890 Reddit posts that were generated by 14,401 authors from March 14 to December 16, 2020. Using active learning and intensive manual inspection, 292 (2.03%) active authors, who were infected by COVID-19 and frequently reported disease progress on Reddit, along with their 2213 (9.67%) longitudinal posts, were identified. Machine learning tools to extract biomedical information were applied to identify COVID-19 symptoms mentioned in the Reddit posts. We then examined longitudinal changes in individual physiological and psychological characteristics before and after recovery from COVID-19 infection.

**Results:**

In total, 58 physiological and 3 psychological symptoms were identified in social media before and after recovery from COVID-19 infection. From the analyses, we found that symptoms of patients with COVID-19 lasted 2.5 months. On average, symptoms appeared around a month before recovery and remained for 1.5 months after recovery. Well-known COVID-19 symptoms, such as fever, cough, and chest congestion, appeared relatively earlier in patient journeys and were frequently observed before recovery from COVID-19. Meanwhile, mental discomfort or distress, such as brain fog or stress, fatigue, and manifestations on toes or fingers, were frequently mentioned after recovery and remained as intermediate- and longer-term sequelae.

**Conclusions:**

In this study, we showed the dynamic changes in COVID-19 symptoms during the infection and recovery phases of the disease. Our findings suggest the feasibility of using social media data for investigating disease states and understanding the evolution of the physiological and psychological characteristics of COVID-19 infection over time.

## Introduction

COVID-19 is a pandemic viral infectious disease that has quickly spread worldwide. The clinical presentation of COVID-19 has been well defined from active clinical and basic scientific research. Risk factors and commonly observed symptoms at the diagnosis of COVID-19 and throughout the acute disease course have been well documented [1,2]. Several studies have tried to identify self-reported COVID-19 symptoms using social media data [3,4] and investigated the dynamics of symptoms that were observed prior to and throughout COVID-19 infection [5]. Our own analysis of social media and clinical literatures suggested less common or rarely observed novel symptoms related to COVID-19. We also observed that different sets of clinical and demographic characteristics are associated with specific clinical outcomes, such as severity of disease progression, hospitalization, and intensive care unit admission [6]. These efforts have helped to understand the development of COVID-19 and identify appropriate medical treatment options and diagnostic methods.

Since early 2021, researchers have found emerging evidence of long-term sequelae in a considerable proportion of patients who have recovered from COVID-19. Several systematic reviews and cohort-based studies have suggested a set of symptoms from which COVID-19 survivors have partially recovered or those they have retained [7,8]. Most of these studies have focused on a set of symptoms that appeared at a static time point (eg, symptoms before, or at, the moment of confirmation of COVID-19; symptoms during recovery; or symptoms after recovery from COVID-19) and were based on retrospective data of hospitalized patients. When we considered that about 15% of patients with COVID-19 have been hospitalized [9], there was limited understanding of patients who experienced less severe symptoms and received home-based care. This created a huge knowledge gap to understand the symptom landscape and symptom durations in the general patient population (ie, 85% of patients with COVID-19). A comprehensive understanding of the full spectrum of symptoms and their dynamic changes throughout the patient journey (ie, disease course) was essential to obtain better information about acute and chronic/persistent COVID-19 symptoms in general patients and develop a complete understanding of the longitudinal impact of COVID-19 infection in the population.

To better understand the longitudinal changes in the physiological and psychological characteristics of the COVID-19 patient population throughout the patient journey, we systematically investigated Reddit public social media data from individuals who had previously been infected with COVID-19 and have recovered. Social media provides an efficient method of gathering large amounts of real-world data on the general public, which are scalable and convenient for users at any time of day, especially from remote or unattended regions. In addition, the development of sophisticated machine learning methods has been used to collect high-quality medical information from social media [10,11]. For this study, social media data were utilized to capture the disease course of general patients with COVID-19, including nonhospitalized patients with various levels of symptom severity. Machine learning methods were applied to select reliable patients with COVID-19 and their posts from social media data and to identify the full spectrum of symptoms of COVID-19 in a comprehensive manner.

## Methods

### Data

Reddit was used to collect social media posts of COVID-19 survivors. Reddit is a large user-generated content website and text-based social media platform (without the limitation of text length) enabling us to extract detailed information about a given topic. Reddit is also a community-based social media platform providing sections devoted to topic-specific discussions (subreddits). Authors express their opinions or concerns on subreddit forums. Subreddit defines its characteristics and eligible members. Therefore, people who are really interested in each topic can participate in a subreddit community. Furthermore, Reddit provides a publicly accessible Application Programming Interface (API) so that researchers can collect and analyze anonymized posts for their own purpose. Such clear definitions of topics and member eligibility, as well as anonymized data access through the API, made Reddit a reliable and effective social media platform to find the appropriate target audience for this study.

For this analysis, we collected posts that were generated from the moment the United States declared COVID-19 a national emergency (March 13, 2020; we collected posts from March 14, 2020) until the Food and Drug Administration (FDA) issued an emergency use authorization to both Pfizer-BioNTech’s and Moderna’s COVID-19 vaccines (December 17, 2020; we collected posts until December 16, 2020). After the World Health Organization (WHO) declared COVID-19 a pandemic and the United States declared COVID-19 a national emergency, it was logical to assume that the general population started to become aware of the presence of the COVID-19 virus and be interested in symptoms and their health conditions. In addition, they were exposed to the risk of COVID-19 contraction before the vaccine was available. During this period, people could actively discuss COVID-19-related issues and share their personal experience. We especially considered posts generated in “COVID-19Positive” and “CoronavirusSurvivors” subreddits. “COVID-19Positive” was self-described as “a place for people who came back positive for COVID-19 to share your stories, experiences, answer questions and vent!” “CoronavirusSurvivor” was self-described as a community for survivors of the coronavirus. At the time the conversations analyzed for this study were downloaded, the “COVID-19Positive” subreddit had about 81,000 members and the “CoronavirusSurvivors” subreddit had about 1000 members.

**Figure 1:**
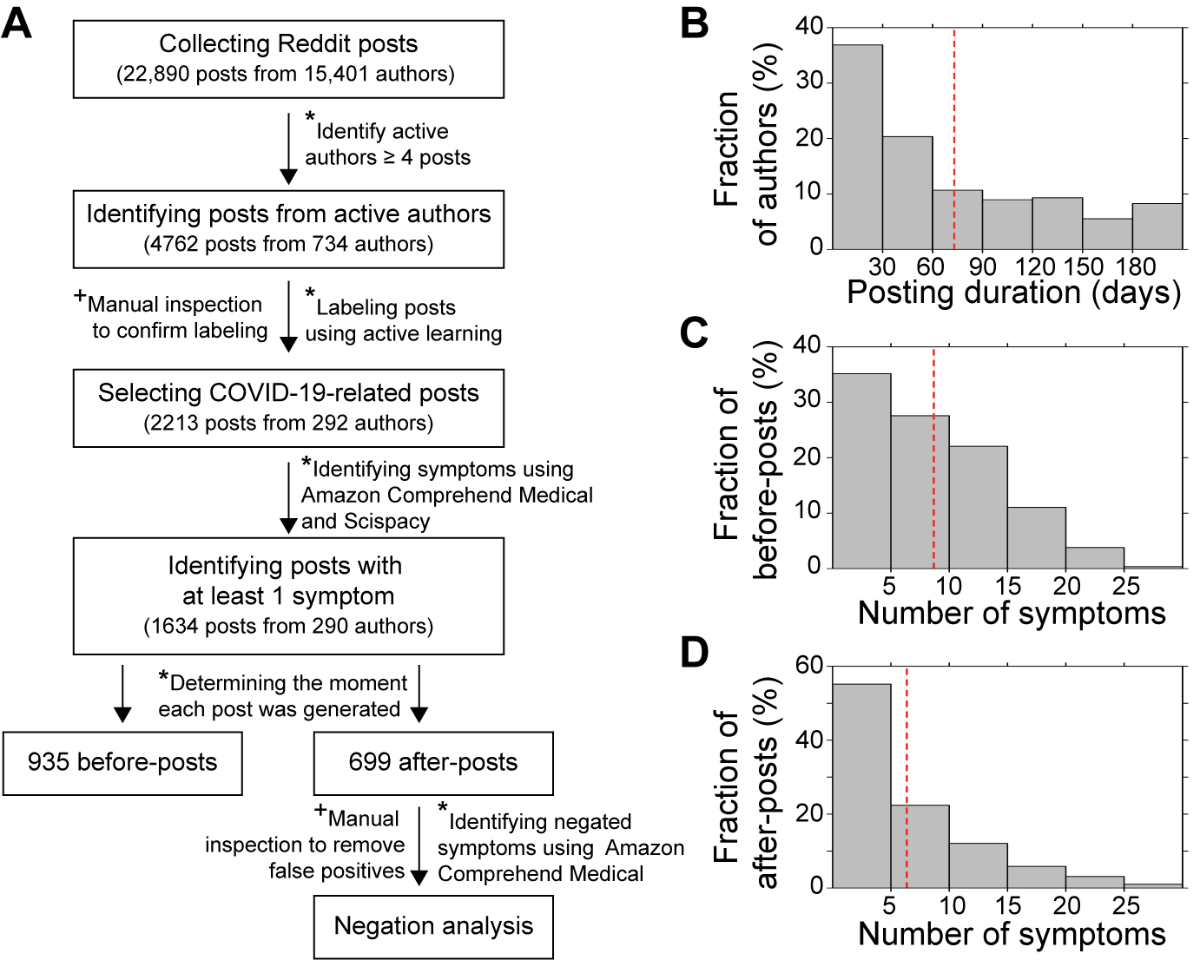
Overview of social media data related to COVID-19 patient journey. (A) Social media data collection and analysis procedure. In total, 1634 posts from 290 active authors who continuously posted their disease progress were identified and used for further analyses. * and + indicate automated steps and steps requiring manual inspections, respectively. (B) Fraction of active authors, depending on posting duration. Posting duration is the time difference between the first and last COVID-19 posts from the same active author. The red-dashed line indicates the average posting duration. (C) Fraction of before-posts, depending on number of symptoms. (D) Fraction of after-posts, depending on the number of symptoms. The red-dashed line indicates the average number of symptoms mentioned in a post.

The procedure to process and analyze social media data is described in Figure 1A. In total, 22,890 posts from 15,401 authors were collected from Reddit. Of the 15,401 authors, 11,900 (77.27%) generated only 1 post, which was inappropriate to examine longitudinal changes in COVID-19 symptoms. We did not consider them for further analyses.

To identify active authors’ authentic COVID-19-related posts, we performed active learning. After the automated active learning procedure, manual inspection was performed to examine the reliability of post labeling (Figure 1A). Details are described in the Acquiring Pertinent Labels for Reddit Posts section.

### Acquiring Pertinent Labels for Reddit Posts

We observed some authors asking questions about COVID-19 or posting about symptoms, or about a family member, rather than themselves. It was apparent after reading many posts that not all authors were COVID-19 positive. This observation necessitated distinguishing between true patients with COVID-19 and authors who had not tested positive for COVID-19. Furthermore, we wanted to identify those authors that had discussed recovery from COVID-19 in their posts. We were also interested to know how different physical and psychological symptoms were discussed within Reddit posts.

We first generated an unlabeled data set of 4762 posts that were written by 734 authors who had posted at least 4 times in our COVID-19-related subreddits (Figure 1A). We designated 4 classes (ie, label types) that we needed for our analysis. For each post in our data set, we needed to check whether the post had (1) evidence that the author is/was COVID-19 positive (label: “posi”), (2) evidence that the author has recovered from COVID-19 (label: “reco”), (3) evidence of physiological symptoms (label: “physio”), and (4) evidence of psychological symptoms (label: “psycho”). More details of labeling are described in Multimedia Appendix 1.

To do this, we implemented an active learning method [12] to capture the different labels for Reddit posts in an automated and expedited manner. Active learning is a popular method to find relevant materials from within documents and is widely used in the e-discovery domain (eg, labeling documents as relevant or nonrelevant from a collection of legal documents or research papers). It also has been applied to identify the literature that is relevant to infection prevention and control of COVID-19 from a large biomedical corpus [13] and used to build a diagnostic method for COVID-19 [14].

Using active learning, we built a classifier for each of the label classes; the classifier was periodically updated with new evidence, as saved by a human assessor (Multimedia Appendix 1). This way, the classifier can capture newly found text features with each training iteration. The goal for the classifier is to find the next best-candidate Reddit post that can contain a label evidence string. The human assessor, when presented with the candidate post, assesses the post as nonrelevant (providing a true-negative label) or highlights and saves an evidence string (providing a true-positive label). After retraining with a set of new labels, the classifier “relearns” which features in the text are likely to be found relevant and it presents the next best candidate to the user for assessment. For the human assessor, the likelihood of finding true-positive labels increases early in the label-gathering process, which helps with expediting labeling efforts when we are looking for true-positive labels on a budget.

A labeling interface was built to capture labels using our active learning method (Multimedia Appendix 1). An assessor is presented with 1 Reddit post at a time for 1 label class. The assessor can mark the post as nonrelevant for the particular post for the particular class or save (copy/paste) an evidence string that is representative of the label class description. The classifier for the respective label type is then retrained with the new evidence string. Then, all remaining unlabeled posts are scored by the classifier. The top-ranked unlabeled post is then presented to the assessor for judgement. Thus, after each judgement cycle, the classifier learns which text features are relevant to the assessor, and the method then presents the most likely relevant candidate for assessment to the user next. This way, the most relevant posts are labeled quickly, with the added advantage that assessment budgets and available time are utilized more efficiently.

We recruited 13 well-trained assessors for the labeling task; the number of assessors varied across label classes. The goal for assessors was to find and label as many label-relevant posts as fast as possible. To do this, we provided 2 hours of training sessions so that accessors could find relevant posts generated by reliable patients with COVID-19. Labeled data completed by accessors were then manually inspected by the authors in this study to confirm that labeling was done correctly (Multimedia Appendix 1). The assessors made 2785 judgements and found 1072 evidence strings across all classes (Multimedia Appendix 1).

### Identifying Active Authors

We selected active authors who were infected by COVID-19 and described their experiences and COVID-19 symptoms across the entire patient journey: from the confirmation of COVID-19 infection to after recovery from COVID-19. To do this, we examined the median time duration between the first post and the last post that a given user generated (Multimedia Appendix 1), and selected the optimal number of posts that could represent the patient journey.

We found that symptoms usually take 5-6 days to appear after COVID-19 exposure (incubation period of COVID-19) [15]. We also estimated the recovery time after onset of the symptoms. We calculated the average recovery time (duration between the posts with the label “posi” and the posts with the label “reco”) for the subset of users who had mention of recovery in their posts. For this calculation, first we removed the outliers that were considered as cases with recovery time higher than 2×SD. We obtained 15.7(SD 10.4) days as the average recovery time after testing positive. Since our subset was small (n=38 users) and there was high variation in the data, we decided to use the commonly accepted recovery time from the literature. Although there were various studies reporting different durations [5], 14 days was the duration we most often found in our search [16-19]. Therefore, we utilized 14 days as the recovery period after having tested positive for COVID-19, and we assumed that 20 (6+14) days of posting period could represent a typical patient’s journey. In addition, 4 posts were used as a cut-off to find active authors since they were generated for 22 days, which was the closest period to our adopted notion of COVID-19 duration (20 days).

### Identifying Symptoms of COVID-19

To identify the physical or psychological symptoms that were mentioned in Reddit posts, we applied 2 automated symptom extraction methods. Using the Amazon Comprehend Medical tool (Amazon Web Services), we extracted medical entities. The Amazon Comprehend Medical tool uses machine learning to extract health-related information from text automatically. For this study, we considered medical entities “symptoms” and “signs” as COVID-19 symptoms. In addition to the Amazon Comprehend Medical tool, we also built a model to identify medical entities using Scispacy (v.0.4.0). Scispacy is a Python package for handling scientific documents and extracting medical and clinical terminology [20]. We considered the medical entity “disease” as COVID-19 symptoms. From the performance evaluation of medical entity extraction models, we found that the models identified over 80% of COVID-19 symptoms correctly and reliably (Multimedia Appendix 1). The model achieved 87% precision, 83% recall, and an F1 score of 0.85. In total, 58 physical and 3 psychological symptoms were identified (Multimedia Appendix 1). The 3 psychological symptoms were “confusion or fluster,” “depression or anxiety,” and “mental discomfort or distress” (eg, foggy head and loss of consciousness).

In total, 1634 posts generated by 290 active authors mentioned at least 1 COVID-19 symptom (Figure 1A). Next, we determined when each post was generated: before or after recovery from COVID-19. We divided the posts into 2 groups, before-posts and after-posts, based on their posting time. Before-posts were written before COVID-19 recovery, and after-posts were written after recovery. Posts that were generated before recovery posts (label: “reco”) were defined as before-posts. When authors only had positive posts (label: “posi”), posts generated from the date of the first positive post to the next 14 days were considered as before-posts based on the study that patients with COVID-19 take about 14 days to recover from the disease [16]. Remaining posts were defined as after-posts. We assumed the date of the first before-post was the moment users realized COVID-19 symptoms or confirmed their infection by COVID-19.

Since the number of before- and after-posts was different, identified COVID-19 symptoms were observed with different frequencies. For a fair comparison of symptom frequency between before and after COVID-19 recovery, we normalized the frequency of each symptom to 100 posts (ie, of 100 posts, how many mentioned a given symptom). Depending on this frequency, acute and chronic symptoms were determined. Acute symptoms developed rapidly and were mentioned more frequently in before-posts. Chronic symptoms developed gradually and were slow to resolve, remaining as sequelae, and were mentioned more frequently after recovery from COVID-19 (after-posts).

### Identifying Symptoms Mentioned Together

To identify symptoms that commonly appear together, we performed association rule analysis [21]. We measured support, which is defined as the proportion of posts in which a certain set of symptoms come together. We adopted the Apriori algorithm and followed a bottom-up approach that starts from every single symptom, and then symptom subsets are extended 1 item at a time. At each step, the group of candidates is tested, and the ones that include infrequent items are pruned.

### Negation Analysis

To understand how patients perceive and respond to COVID-19 symptoms, negation analysis was performed using the Amazon Comprehend Medical negation model [22] and manual inspection (Figure 1A). Negation analysis showed whether a given symptom was denied (eg, “I have gotten no fever” or “I don’t cough anymore”). Therefore, in our study, negation indicated that a given symptom was relieved or disappeared after recovery from COVID-19. After automatic identification of negated symptoms, we removed false positives through manual inspection. False positives are symptoms that present after COVID-19 recovery, but negation analysis detected them due to the sentence structure (eg, “I still have no smell and taste after I received a negative polymerase chain reaction [PCR] test”; “no smell and taste” was not negation. This symptom was still presented after recovery). Of 699 after-posts, negation was detected from 240 (34.3%) posts.

### Statistical Analysis

To compare the duration of symptoms before and after COVID-19, Spearman rank correlation coefficient measurement was performed using the programming language Python (v.3.8.1) and SciPy package (v.1.6.2). *P*<.05 was considered statistically significant. For the visualization of analyses, the BPG library (v.6.0.1) in R was used [23].

## Results

### Result 1: Overview of Reddit COVID-19 Posts

To understand longitudinal changes in physical and psychological characteristics during the COVID-19 patient journey, we decided to collect patients' self-generated posts from Reddit. Reddit is a community-based online forum where patients can share their clinical journey, symptoms, and experiences from diagnosis to postrecovery. We identified 292 active authors who continuously and voluntarily discussed their physiological and psychological symptoms from the beginning of COVID-19 contraction to the after-recovery stage of COVID-19 (see the Methods section and Multimedia Appendix 1 for details). From the active learning experiment, we found that active authors generated 2213 posts that described their positive COVID-19 infection and the symptoms they had before and after recovery from COVID-19. On average, active authors generated 8 posts in 73 days (difference between the first before-post and the last after-post; Figure 1B). It is suggested that COVID-19 symptoms remain after recovery and present for about 2.5 months from diagnosis.

Next, we fed all 2213 posts into the biomedical named entity recognition (Bio-NER) program to find COVID-19 symptoms in a comprehensive manner. In total, 1634 (73.84%) posts mentioned at least 1 COVID-19 symptom (Figure 1A). From those, 935 (57.22%) posts were written from diagnosis (eg, confirmation of a positive test or notification of COVID-19 symptoms) to before recovery from COVID-19 (eg, received a negative test after the positive test). These were defined as before-posts. In addition, 699 (42.78%) posts were written after recovery (after-posts; see the Methods section for details). On average, each active author mentioned 9 symptoms in the before-post (Figure 1C) period and 6 symptoms in the after-post (Figure 1D) period.

### Result 2: COVID-19 Symptom Landscape in Social Media Data

**Figure 2:**
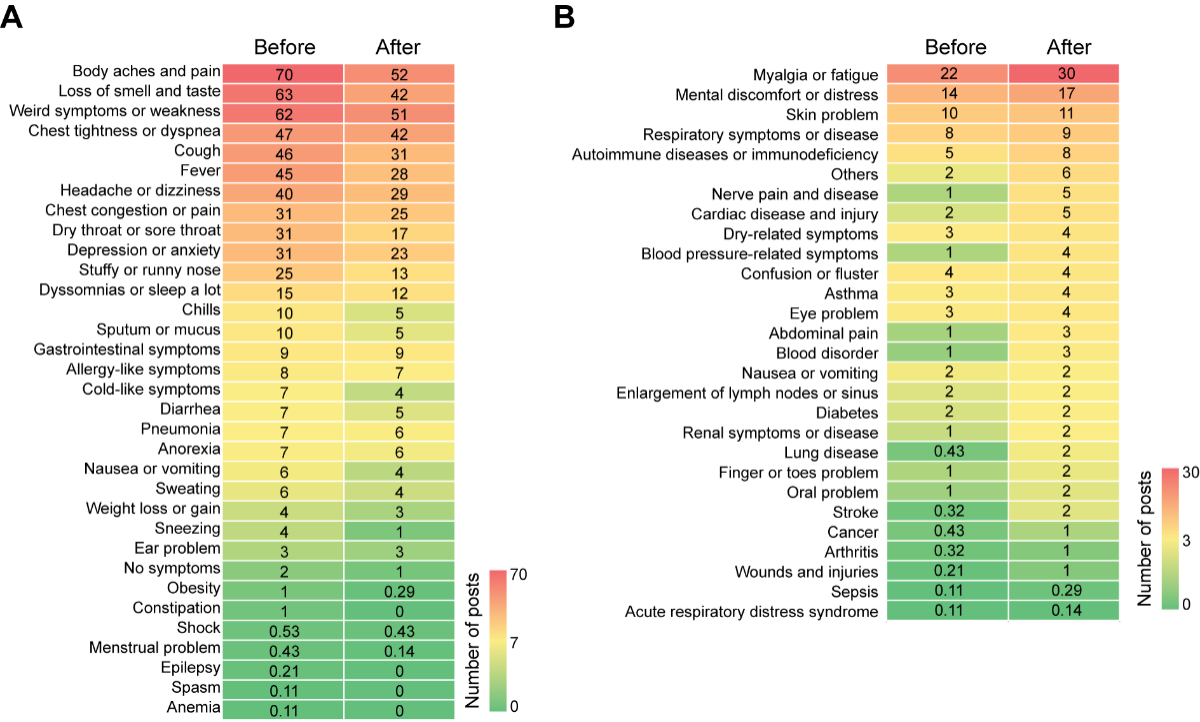
Symptom landscape in before- and after-posts. (A) There were 33 acute symptoms that were frequently mentioned before recovery from COVID-19. (B) There were 28 chronic symptoms that were frequently mentioned after recovery from COVID-19. The number indicates the incidence of a given symptom per 100 posts.

To understand the dynamic development of physiological and psychological symptoms throughout the COVID-19 patient journey, we compared how often individual symptoms were mentioned in before- and after-posts. Of 61 symptoms, 33 (54%) were acute symptoms. They were mentioned more frequently before recovery from COVID-19 compared to after recovery. The most known COVID-19 symptoms, such as weakness, body aches, cough, fever, chest tightness, and loss of smell and taste, were mentioned frequently in before-posts. We observed that these symptoms were relieved or improved after recovery. For example, in 100 posts, loss of smell and taste was mentioned 63 times in before-posts. However, after recovery, it was 32.89% less frequently mentioned (42 times in after-posts). Fever, cough, and throat discomfort (dry or sore throat) were approximately 40% less frequently mentioned after recovery (Figure 2A). Chills (51.23% reduced), cold-like symptoms (42.38% reduced), nausea (40.04% reduced), and sputum (43.52% reduced) were also mentioned less after recovery. Negation analysis supported these findings. When negation was detected in a sentence that contained a symptom, we could assume that a symptom was relieved or disappeared. We performed negation analysis using after-posts and found that common symptoms, including fever, cough, or chest tightness, were denied (eg, “I have no fever or no cough”) in after-posts. In addition, 5%-20% of after-posts clearly mentioned that these symptoms disappeared after recovery from COVID-19 infection (Multimedia Appendix 1). In addition, 4 (7%) extremely rare symptoms (epilepsy, spasm, constipation, and anemia; less than 1% of posts mentioned these symptoms) were only identified before the recovery period (Figure 2A).

Furthermore, 28 (46%) of 61 symptoms were mentioned more frequently after recovery from COVID-19 (chronic symptoms). We observed that patients with COVID-19 mainly complained of psychological symptoms during this time. Mental discomfort and distress, including brain fog, stress, and panic, were mentioned 18.27% more often, and confusion/fluster was mentioned 8.63% more after recovery. Constitutional symptoms, such as fatigue (25.96%), and physiological symptoms, including problems in toes or fingers (eg, tingly hands/feet and toe/finger rash, 41.26%), renal-related problems (eg, frequent urination and kidney pain, 38.43%), and immunodeficiency (eg, autoimmune disease and immune system disorder, 37.94%), were also frequently mentioned after recovery (Figure 2B). These results suggested that well-known symptoms are likely to be acute symptoms and do not remain as postrecovery sequelae of COVID-19 infection. Rather, after recovery, patients experience mental discomfort and symptoms that are less common at or shortly following the time of diagnosis of infection and slow to resolve [6].

### Result 3: Co-occurrence of COVID19 Symptoms Before and After Recovery

**Figure 3:**
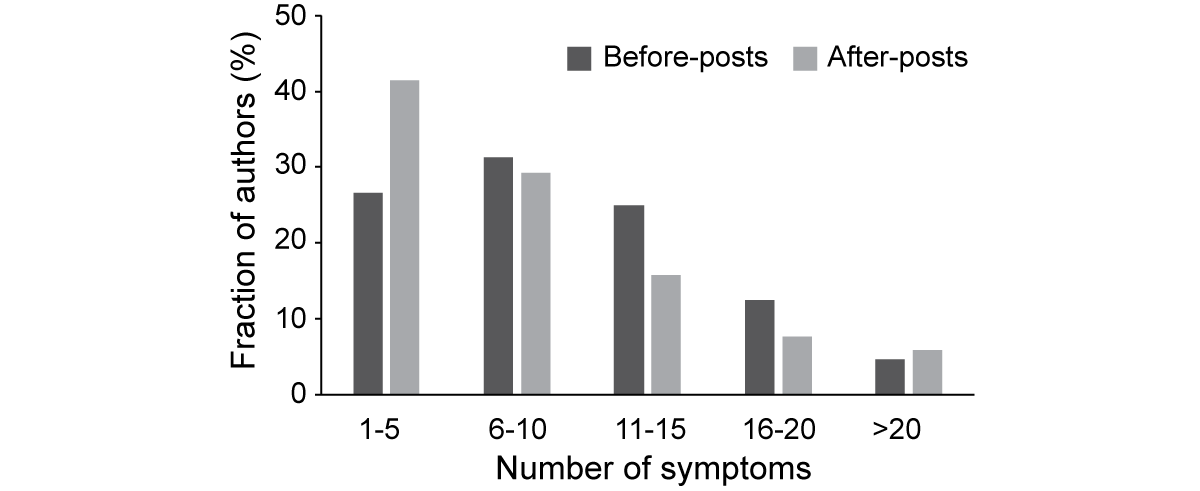
Number of COVID-19 symptoms in before- and after-posts. Dark- and light-gray bars indicate the fraction of authors who mentioned a given number of symptoms in before- and after-posts, respectively.

It has been shown that COVID-19 can cause multiple symptoms during early infection and progression [6]. To understand the dynamic co-occurrence of COVID-19 symptoms through the patient journey, we examined a set of symptoms that were mentioned together in a Reddit post. We found that each author mentioned more symptoms before recovery than after recovery (Figure 3). For example, of 256 authors who generated before-posts, 64 (25%) mentioned 11-15 symptoms, and this was 1.63 times higher compared to those who generated after-posts (n=222; 35 [15.8%] mentioned 11-15 symptoms). Meanwhile, 92 (41.4%) of 222 authors mentioned fewer symptoms (1-5 symptoms) in after-posts, which was 1.56 times higher (68/256, 26.6%) compared to before-posts. Next, we examined what symptom pairs were observed before and after recovery. In total, we identified 368 co-occurred symptom pairs. We observed that common and acute symptoms of COVID-19 were mentioned more frequently together before recovery. Chills with loss of smell and taste (2.51 times), cough (2.44 times), fever (2.39 times), chest tightness (2.39 times), or body aches (2.31 times) frequently co-occurred before recovery from COVID-19 compared to after. Meanwhile, 1 of the chronic symptoms, immunodeficiency (eg, autoimmune disease and immune system disorder), co-occurred with other chronic symptoms, such as mental discomfort/distress, myalgia/fatigue, and skin problems only after recovery from COVID-19 (Multimedia Appendix 1).

### Result 4: Symptom Duration in Patients with COVID-19

**Figure 4:**
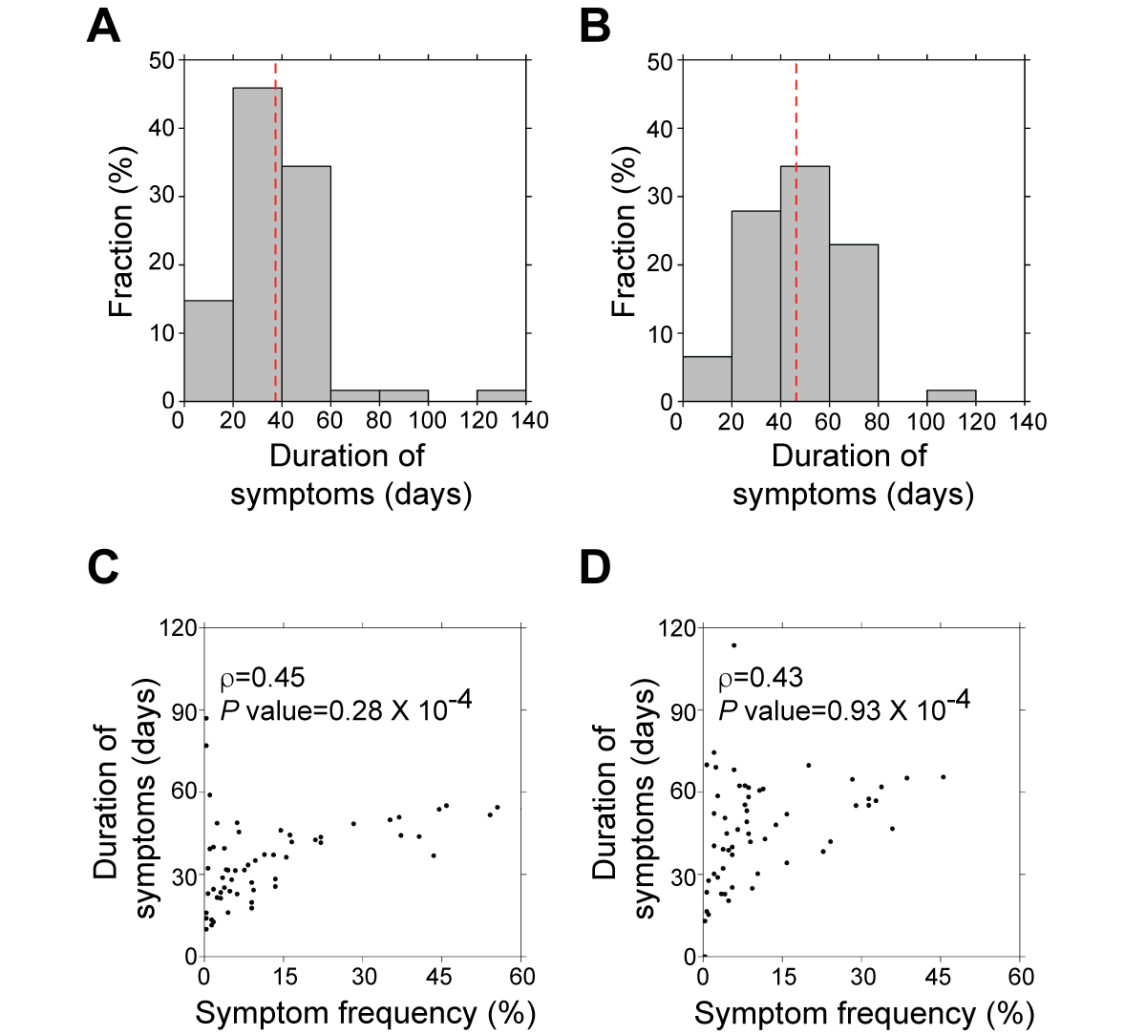
Duration of COVID-19 symptoms. (A) Duration of symptoms before recovery from COVID-19. (B) Duration of symptoms after recovery from COVID-19. The red-dashed line indicates the average symptom duration. The relationship between symptom duration and symptom occurrence (C) before and (D) after recovery from COVID-19.

Our data set was composed of posts that followed time courses, enabling us to perform temporal analyses to understand dynamic changes over time. We examined the duration of symptoms across the COVID-19 patient journey. On average, each symptom persisted for 83 days. On average, symptoms appeared 37 days before recovery (Figure 4A) and remained up to 46 days after recovery (Figure 4B). This implied that about a month was required to recover from the first COVID-19 symptom presentation and that symptoms remained for 1.5 months after recovery.

We found that there was a weak correlation between frequency of symptoms and symptom duration (ρ=0.45, *P*=.28×10^-4^; Spearman correlation coefficient, Figure 4C). In before-posts, well-known acute COVID-19 symptoms (eg, weakness, body ache, cough, fever, dyspnea, and headache) were mentioned by at least 116 (40%) of 290 active authors. They appeared relatively early compared to other symptoms (appeared 54 days before recovery). Loss of smell and taste (37 days), nausea (37 days), and asthma (31 days) appeared about 1 month before recovery. Less common symptoms, including arthritis, epilepsy, and anemia (<1% of before-posts mentioned these) appeared around 10 days before recovery (Multimedia Appendix 1).

After recovery, we observed a similar trend. There was a weak but statistically significant positive correlation between frequency of symptoms and time of presentation (ρ=0.43, *P*=.93×10^-4^; Spearman correlation coefficient, Figure 4D). Chronic symptoms (frequently observed after recovery), such as confusion, immunodeficiency, and fatigue, were slow to resolve. They remained for over 50 days after recovery. Mental discomfort/distress (38 days) also remained longer than other symptoms. Menstrual problems, shock, and acute respiratory distress–related symptoms (<1% of after-posts) remained about 15 days after recovery (Multimedia Appendix 1).

## Discussion

### Principal Findings

Our longitudinal analysis based on social media data showed the dynamic evolution of symptoms through the COVID-19 patient journey. From this observational study, we identified acute and chronic symptoms that were frequently or specifically observed before and after recovery from COVID-19. Individual symptoms showed differing durations and recovery times. Social media data expanded our understanding of COVID-19 symptoms and their longitudinal changes through the patient journey.

From social media data, we observed that the most common COVID-19 symptoms (eg, fever, cough, and weakness) appeared earlier in patient journeys and were mentioned more frequently before recovery from COVID-19. These common COVID-19 symptoms were easily recognizable and evaluated by ordinary people without any screening tools. In addition, they were basic indicators used in clinical and medical research. Accumulated COVID-19 research defined them as well-characterized diagnostic indicators for COVID-19 [24]. Furthermore, at the beginning of the pandemic, national- or international-level awareness campaigns were conducted with a limited understanding of COVID-19 symptoms. We suggested that the public’s basic awareness and easy recognition could disproportionate the prevalence of COVID-19 symptoms, increasing the reporting of common symptoms in social media before recovery from COVID-19.

Interestingly, common symptoms were relieved or disappeared after recovery from COVID-19 (acute symptoms) based on our analyses of symptom negation and the duration over this entire period. Fever was considered a beneficial response to infection. Increased temperature reduces pathogens’ survival and increases mobilization of immune cells [25]. Cough is an intrinsic and protective reaction to many respiratory infections [26]. Skeletal muscle atrophy can be caused by an immune response, leading to weakness or body ache [27,28]. Taken together, we suspected that many common and acute symptoms are likely to be associated with the initial immune response and are relieved as the initial immune response decreases over time after viral clearance.

### Significance of Mining Social Media Data

Currently, there is limited information about nonhospitalized or initially asymptomatic patients with COVID-19 who have had persistent and chronic symptoms after their recovery. In addition, there is a lack of information about longitudinal changes in COVID-19 symptoms due to the limited methods or accessibility to identify COVID-19 survivors on a global level. Our study explored dynamic changes in COVID-19 symptoms throughout patient journeys using social media data. Although social media may lack some depth of patient information, it provides an effective method of collecting a wide breadth of data. Social media data can be easily gathered across the world 24 hours a day, without the need for a clinician visit, and is an extremely efficient method [29] for rapidly disseminating new knowledge related to COVID-19 [30]. Indeed, we observed more than 60 symptoms that were extracted from the Reddit posts, including all the symptoms of COVID-19 suggested by the Centers for Disease Control and Prevention (CDC) [31]. This number of symptoms observed in social media was about 2 times higher than the number of symptoms mentioned in the biomedical literature (34 symptoms), which was published by clinical institutes [6]. Furthermore, tracking COVID-19 symptoms in social media data over time gave us novel insights to understand the full clinical spectrum of symptoms and the patient journey. Social media has also been used to predict COVID-19 waves [32], forecast the number of cases [33], and develop crisis management strategies [34]. Taken together, social media data could be useful for understanding the symptoms and epidemiology of novel diseases, such as COVID-19. Accumulated knowledge and techniques that utilize social media data would be rapidly applied for future pandemic preparedness and response in an effective manner.

### Limitations and Future Work

The presence and rapid diffusion of misinformation in online communities is a growing concern for patients and health care providers [35]. We suspected that similar experiences and common interests among patients would make strong emotional connections in online disease communities, enabling us to extract relatively reliable information compared to nondisease communities. It has been shown that online disease communities are mainly composed of patients, family members of patients, and caregivers who are in similar situations and want to obtain accurate information by posting their real experience-based inquiries online [36]. In addition, patients are more likely to express emotions and share aspects of their life in an online community that they would not share face-to-face [37]. Such similar experiences, common interests, emotional connections, and empathy could develop and operate patient empowerment by creating and disseminating relevant information that would help them to understand their health conditions and to receive psychological support. Furthermore, disease communities develop practices that improve the quality of the information that peers exchange [38]. Since disease communities deal with life-related matters, peer interactions in online disease communities have found low levels of inaccuracy [39], and most false or misleading statements are rapidly corrected by participants [40]. In addition to patients’ honest voluntary participation, we adopted an active learning method by collecting manually labeled Reddit posts and performed second-round manual inspections of labeled posts to improve the authenticity of the social media data. We believe these efforts have greatly improved the reliability and authenticity of our study.

It could be possible that all the symptoms extracted from Reddit would not cover the entire literature of COVID-19 symptoms or would not necessarily be COVID-19 symptoms. Moreover, there is an inherent uncertainty in social media analysis about the accuracy of temporal directions and the potential delays that might occur between the actual event and the time of posting, which is challenging to detect. A closely related limitation is our assumption of a duration of 20 days for the COVID-19 patient journey, which is derived through the available data (Reddit posts).

Natural language processing (NLP) could assist in identifying the temporal mentions and adjusting the outcomes. We might be able to improve our recovery time estimates using NLP, helping to improve temporal accuracy. As we work beyond social media surveillance and integrate with other data sets in the future, we are likely to find better alignment regarding the patient journey and related timelines.

We believe that the integration of various social media data sets and the accumulation of data or systematic surveys of COVID-19 survivors as well as customized machine learning algorithms to capture COVID-19 symptoms would help to decide which symptoms are manifestations of COVID-19 infection and provide new and untapped insights into understanding the longitudinal evolution of symptoms throughout the entire COVID-19 patient journey. This also could be a novel means of assessing the fraction of COVID-19 survivors with persistent symptoms.

### Conclusion

In this observational study, we demonstrated the extensive variability of physiological and psychological impacts of COVID-19 infection and their variability during the acute infection and recovery phases of illness. We also demonstrated the usefulness of gathering social media data as an effective and alternative way to understanding the patient journey from diagnosis through recovery. Our findings show the practicality and feasibility of employing social media data for investigating disease states and understanding the evolution of physiological and psychological characteristics of disease over time. These practices could be incorporated into routine procedures for COVID-19 patient care, providing appropriate treatment and long-term care after recovery from COVID-19.

## Appendix

Multimedia Appendix 1 Supplementary methods, figures, and tables.

## Abbreviations

API: Application Programming Interface
NLP: natural language processing
