# Supplementary material for: Longitudinal Changes of COVID-19 Symptoms in Social Media: Observational Study
Source: J Med Internet Res. 2022 Feb 16;24(2):e33959. doi: 10.2196/33959 (PMC8852652; doi:10.2196/33959)
Supplement: Multimedia Appendix 1 [file jmir_v24i2e33959_app1.docx]

**Supplementary Methods, Figures and Tables**

**Longitudinal changes of COVID-19 symptoms in social media: Observational study**

### Sarah Sarabadani^1^, Gaurav Baruah^1^ , Yan Fossat^1^ , Jouhyun Jeon^1, *^

^1.^ Klick Applied Sciences, Klick Inc., 175 Bloor Street East, Suite 300, Toronto, Ontario, Canada, M4W 3R8.

**Multimedia Appendix 1.** Identification of COVID-19 patients and their post using active learning, and performance evaluation of medical entity extraction models.

**Supplementary Figure 1.** Time differences between the first and the last posts depending on the number of posts.

**Supplementary Figure 2.** Labelling interface to capture evidence for “posi”, “reco”, “phsyio”, and “psycho” label types.

**Supplementary Figure 3.** Negation of symptoms after the recovery of COVID-19. The fraction of after posts which mentioned relief or disappearance of a given symptom was shown.

**Supplementary Table 1.** 1,072 evidence strings that were found for posi, reco, physio, and psycho label types using our active learning method.

**Supplementary Table 2.** 61 symptoms identified from Reddit posts.

**Supplementary Table 3.** Occurrence of symptom pairs before and after COVID-19 recovery.

**Supplementary Table 4.** Symptom duration before and after COVID-19 recovery.

**Multimedia Appendix 1.**

**Acquiring pertinent labels for Reddit posts.**

From the Reddit, we initially collected 22,890 posts from 15,401 authors. We only considered 734 authors who generated at least 4 posts during the test period (4,762 posts), and divided posts into 4 groups (4 types of labels). For each post in our dataset we needed to check whether the post has:

- evidence that the redditor is/was COVID-19 positive? (label: **posi**)
- evidence that the redditor has recovered from COVID-19? (label: **reco**)
- evidence of physiological symptoms? (label: **physio**)
- evidence of psychological symptoms? (label: **psycho**)

There were many variations in the way these classes were represented in the Reddit posts. Furthermore, posts could contain evidence of multiple classes. **Supplementary Method Table A** lists examples of each of these classes as found in Reddit posts.

**Supplementary Method Table A.** Examples of evidence strings for our class labels as found in Reddit posts by assessors.

| **Class / Label type** | **Examples of evidence for label found in various Reddit posts** |
| --- | --- |
| Positive (**posi**) | “I am a positive patient”, “Tested positive 24th March”, “Received positive result on Friday morning”, “I am positive as of last week”, “I tested positive”, “my positive COVID test”, “I have COVID-19”, “my positive COVID diagnosis”... |
| Recovered (**reco**) | “Tested negative”, “I got negative”, “Diagnosed and recovered!”, “my doctor officially cleared me from self-isolation”, “I tested positive March 31st and got a negative retest result today” |
| Physiological symptoms (**physio**) | “swollen right neck lymph, And a fever”  “Head splitting headacheGI tract issues nausea, no appetite, and slight diarrhea. Noticed my chest started feeling congested”  “Fatigue; Scratchy throat; fever; chills; Diarrhea; No appetite; heart race; burning feeling in my chest; Awful acid reflux; chest tightness”  “fatigued; sever headache, body aches, chills; insomnia; taste and smell stopped completely; nausea;” |
| Psychological symptoms (**psycho**) | “more anxious; really nervous”  “I’m honestly terrified and just wish I could feel normal again”  “feelings of dread and imminent death are constant ; Borderline hysterical from stress”  “Depression and anxiety caused by the virus” |

**Identification of positive COVID-19 patients and their posts**

To find posts generated by reliable COVID-19 patents and get the reliable labels for positive, recovered, and physiological and psychological symptoms, we performed active learning with 13 human accessors.

Active learning is a popular method to find relevant materials from within documents, and is widely used in the e-discovery domain (*e.g.*, labelling documents as relevant or non-relevant from a collection of legal documents or research articles). Due to its popularity and broad applications, many benchmark or comparison studies have evaluated and proved its efficiency and robustness. For instance, Cormack et.al., showed that active learning required significantly less human review effort than random selection in order to label documents as relevant or not, on eight legal review tasks [1]. Active learning yielded matches (*i.e.,* relevant and related texts or answers) more quickly than simply choosing a randomly selected document for assessment [2]. Other studies have shown that active learning consistently outperformed other methods in various test situations [3, 4]. In pathology and clinical domain, active learning has been successfully applied to identify literature which are relevant to infection prevention and control of COVID-19 from a large biomedical corpus [5], and used to build the diagnostic method of COVID-19, and the study achieved over 90% of diagnosis accuracy [6].

We performed a brief evaluation of active learning by comparing the actual time that accessors spent to label 4,762 Reddit posts with the anticipated time to label these posts. In our study, accessors spent 43.15 hours to complete post labeling, which is 3.68 ~ 7.36 times faster than the anticipated time (158.73 ~ 317.47 hours). Based on the assumption that each post takes 30 seconds ~ 1 minute to label one of “posi”, “reco”, “physio”, or “psyco”, anticipated times are measured (30 seconds × 4 labels × 4,762 posts = 158.73 hour and 1 minutes × 4 labels × 4,762 posts = 317.47 hours). Based on the generally supported efficiency, reliability, widely used applications (including COVID-19), and our brief evaluation of active learning, we presumed the equivalent efficiency of active learning was achieved in our study, and decided to adopt this method to find relevant social media posts that can represent COVID-19 patient journey.

The 13 accessors were recruited from Klick Inc., a technology, media, and research company in the healthcare sector based in Toronto, Canada. They were all employees of Klick Inc. and volunteered via the company’s intranet system. Due to the characteristics of the company and their professional expertise, they all have basic knowledge on biology and healthcare, and are highly interested in pandemic diseases. For the professional assessment, we provided 2 hours of training sessions before they labeled Reddit posts. Sessions were composed of brief introduction of (1) the study (0.5 hour), (2) labeling methods (1 hours), and (3) web-interface to capture labels using our active learning method (0.5 hour). During the labeling methods session, we introduced how to identify real COVID-19 patients by presenting several expressions that represent positive COVID-19 patients (*e.g.,* “my positive COVID test”, “my positive COVID diagnosis”, “I am positive for COVID”, “I tested positive for COVID” and “I have COVID-19” in posts). Assessors also practiced identifying positive COVID-19 patients using 30 COVID-19 related posts (they were only used for training, and were not used for our study). Labeled data done by assessors was further manually inspected by the authors in this study to validate whether posts were generated by COVID-19 patients or not.

**Performance evaluation of medical entity extraction models.**

To evaluate the performance of the entity extraction models, we first manually curated 70 symptoms from a subset of 20 randomly selected Reddit posts. These manually curated symptoms were compared with the symptoms extracted by both AWS Comprehend Medical and Scispacy. Of 70 symptoms, 58 (82.86% of accuracy) were correctly extracted by entity extraction models. The model achieved 87% precision, 83% recall and 0.85 of F1 score. Based on our performance evaluation, we concluded that medical entity extraction models can identify over 80% of COVID-19 symptoms correctly and reliably. Interestingly, we observed that the models correctly identified the symptoms stated as a phrase (*e.g.,* “sense of taste / smell gone”, “breathing capacity is diminished”, “shortness of air”, and “cannot smell or taste anything”). However, the models failed to extract correct symptoms when (1) the content of the phrase was informal or used metaphor (*e.g.,* “everything tastes and smells like rotten onions”, “I gag literally when I breathe”) or (2) typos were in sentences (*e.g.,* lite headed).

**References**

1. Cormack GV, Grossman MR. Evaluation of machine-learning protocols for technology-assisted review in electronic discovery. SIGIR '14: Proceedings of the 37th international ACM SIGIR conference on Research & development in information retrieval; New York, NY, USA2014. p. 153-62.

2. Baruah G, Haotian Z, Rakesh G, Jimmy L, D. SM, Olga V. Optimizing nugget annotations with active learning. In Proceedings of the 25th ACM international on conference on information and knowledge management2016. p. 2359-64.

3. Cormack GV, Grossman MR. Autonomy and Reliability of Continuous Active Learning for Technology-Assisted Review. ArXiv. 2015;abs/1504.06868.

4. Cormack GV, Grossman MR. Scalability of Continuous Active Learning for Reliable High-Recall Text Classification. CIKM '16: Proceedings of the 25th ACM International on Conference on Information and Knowledge Management2016. p. 1039–48.

5. Rios P, Radhakrishnan A, Williams C, Ramkissoon N, Pham B, Cormack GV, et al. Preventing the transmission of COVID-19 and other coronaviruses in older adults aged 60 years and above living in long-term care: a rapid review. Syst Rev. 2020 09 25;9(1):218. PMID: 32977848. doi: 10.1186/s13643-020-01486-4.

6. Wu X, Chen C, Zhong M, Wang J, Shi J. COVID-AL: The diagnosis of COVID-19 with deep active learning. Med Image Anal. 2021 02;68:101913. PMID: 33285482. doi: 10.1016/j.media.2020.101913.

**Supplementary Figure 1.** Time differences between the first and the last posts depending on the number of Reddit posts.

**
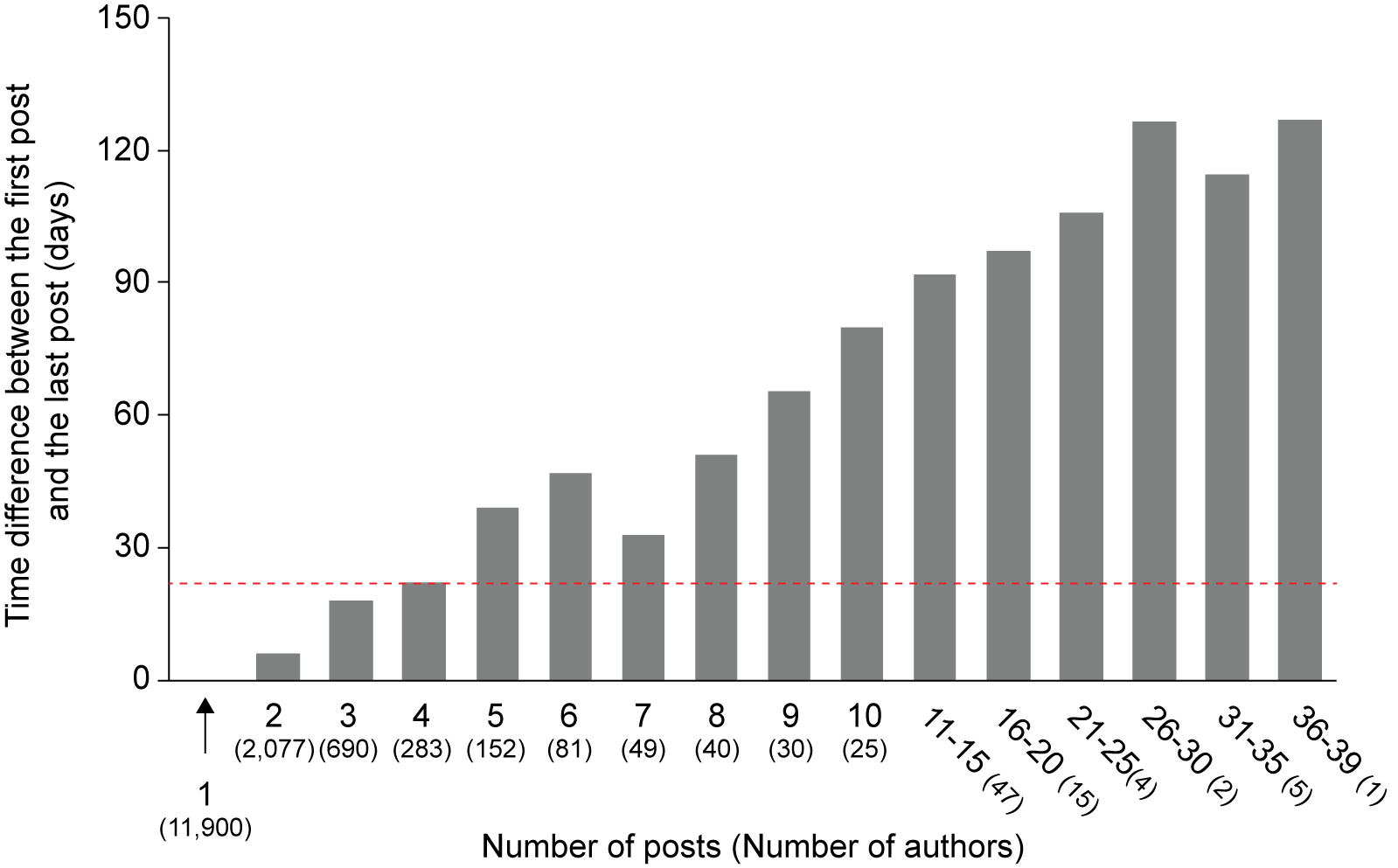
**

* Red dashed line indicated the time duration (22 days) to generate four Reddit posts.

**Supplementary Figure 2.** Labelling interface to capture evidence for “posi”, “reco”, “phsyio”, and “psycho” label types.

**
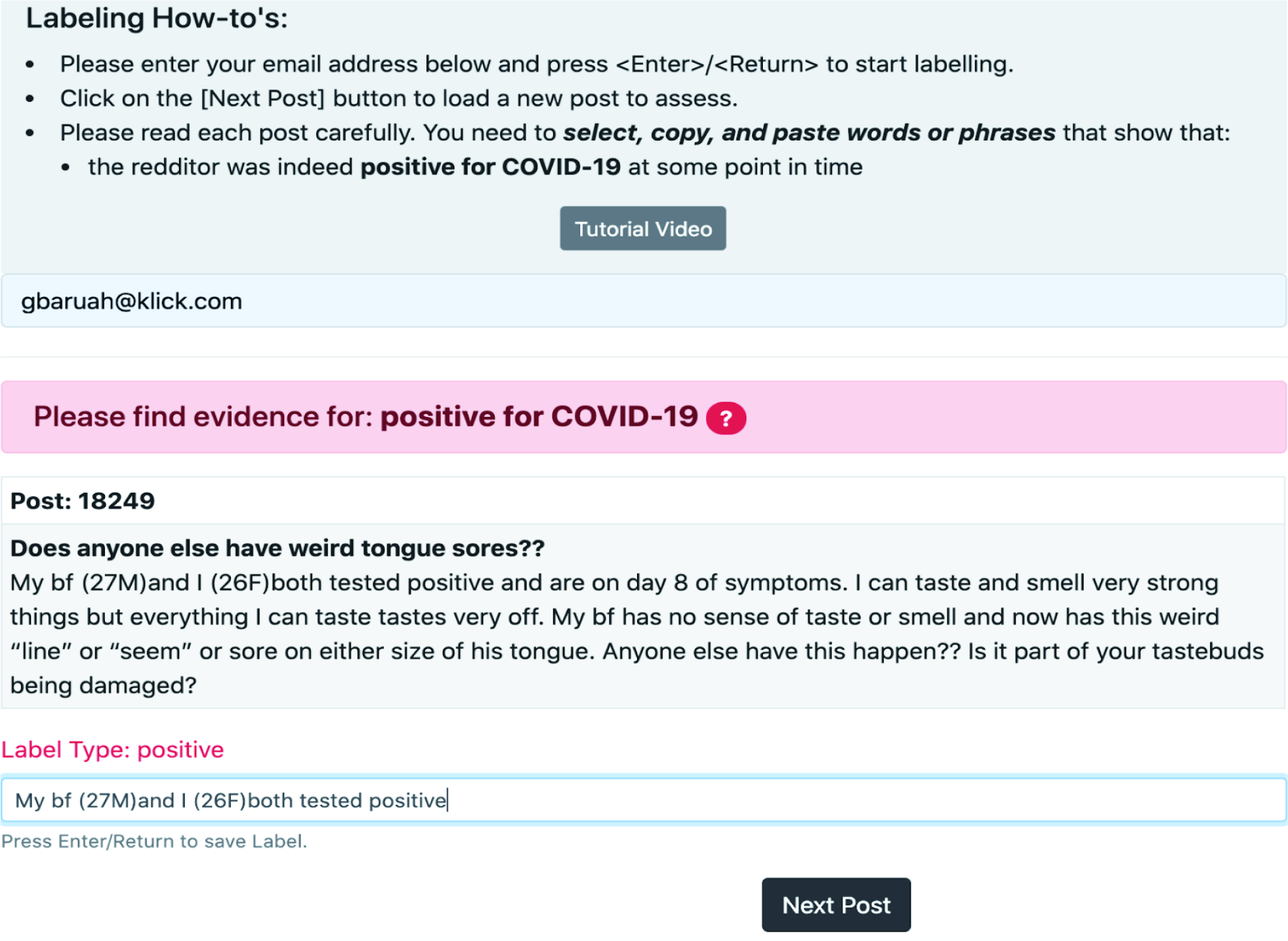
**

**Supplementary Figure 3.** Negation of symptoms after the recovery of COVID-19. The fraction of after posts which mentioned relief or disappearance of a given symptom was shown.


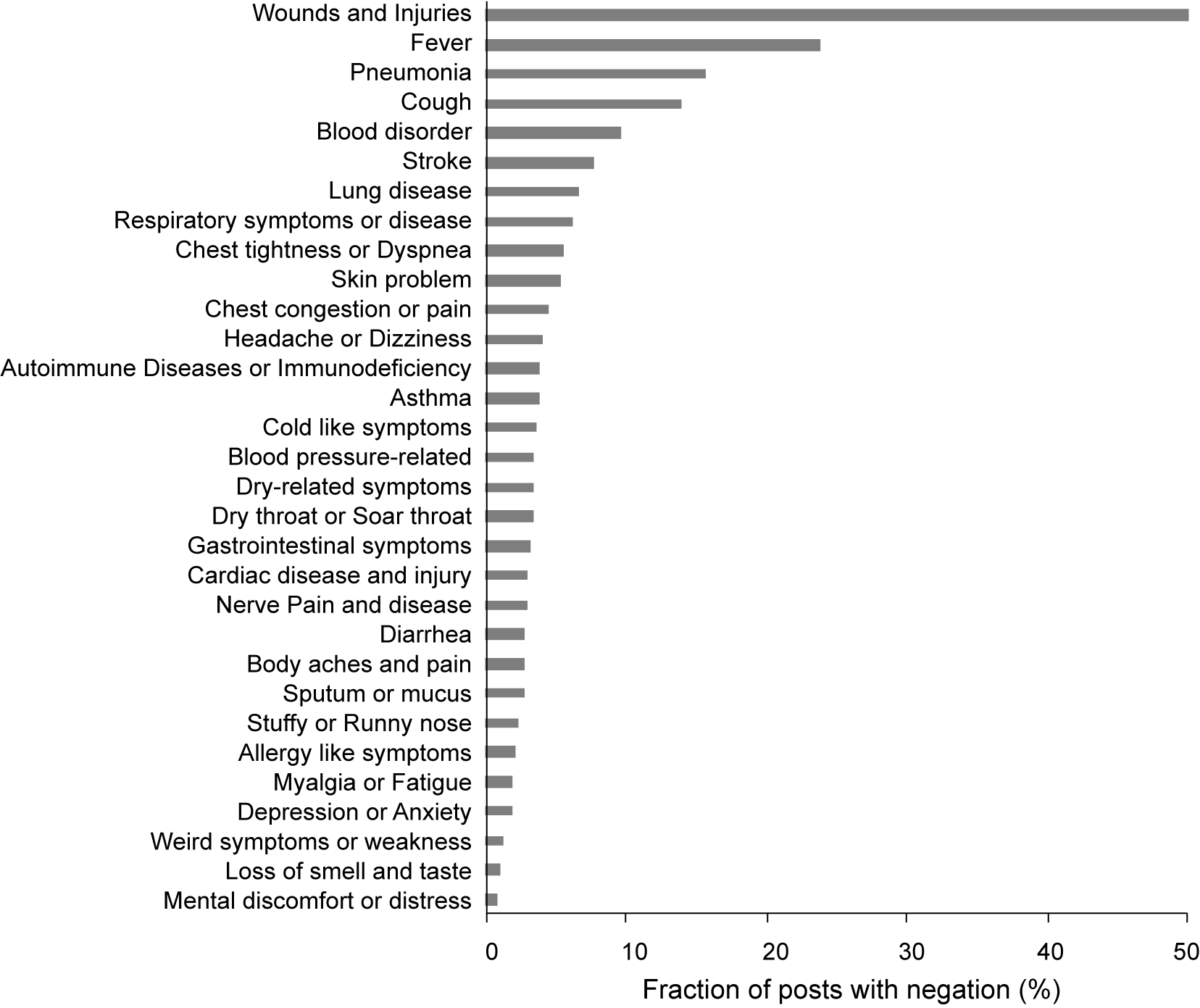


**Supplementary Table 1.** 1,072 evidence strings that were found for posi, reco, physio, and psycho label types using active learning method.

| **label_type** | **n_assessors** | **n_posts_labelled** | **n_relevant_found** |
| --- | --- | --- | --- |
| posi | 7 | 677 | 369 |
| reco | 11 | 1,162 | 293 |
| physio | 4 | 176 | 130 |
| psycho | 13 | 770 | 280 |
| total | 13 | 2,785 | 1,072 |

**Supplementary Table 2.** 61 symptoms identified from Reddit posts.

| **Symptom** | **Number of before-posts** | **Number of after-posts** | **Symptom occurrence per 100 before-posts** | **Symptom occurrence per 100 after-posts** |
| --- | --- | --- | --- | --- |
| Lung disease | 2 | 14 | 0 | 2 |
| Stroke | 3 | 13 | 0 | 2 |
| Nerve Pain and disease | 11 | 35 | 1 | 5 |
| Blood pressure-related | 11 | 29 | 1 | 4 |
| Blood disorder | 8 | 21 | 1 | 3 |
| Abdominal pain | 10 | 23 | 1 | 3 |
| Others | 19 | 42 | 2 | 6 |
| Cardiac disease and injury | 16 | 34 | 2 | 5 |
| Cancer | 4 | 8 | 0 | 1 |
| Sepsis | 1 | 2 | 0 | 0 |
| Oral problem | 9 | 13 | 1 | 2 |
| Arthritis | 3 | 4 | 0 | 1 |
| Mental discomfort or distress | 67 | 86 | 7 | 12 |
| Finger or Toes problem | 11 | 14 | 1 | 2 |
| Renal symptoms or disease | 14 | 17 | 1 | 2 |
| Autoimmune Diseases or Immunodeficiency | 44 | 53 | 5 | 8 |
| Dry-related symptoms | 26 | 30 | 3 | 4 |
| Diabetes | 16 | 17 | 2 | 2 |
| Myalgia or Fatigue | 205 | 207 | 22 | 30 |
| Acute respiratory distress syndrome | 1 | 1 | 0 | 0 |
| Enlargement of lymph nodes or sinus | 17 | 17 | 2 | 2 |
| Asthma | 29 | 27 | 3 | 4 |
| Respiratory symptoms or disease | 71 | 66 | 8 | 9 |
| Eye problem | 29 | 25 | 3 | 4 |
| Wounds and Injuries | 7 | 6 | 1 | 1 |
| Nausea or vomiting | 20 | 17 | 2 | 2 |
| Skin problem | 90 | 75 | 10 | 11 |
| Confusion or Fluster | 33 | 27 | 4 | 4 |
| Gastrointestinal symptoms | 87 | 65 | 9 | 9 |
| Pneumonia | 62 | 45 | 7 | 6 |
| Anorexia | 61 | 43 | 7 | 6 |
| Chest tightness or Dyspnea | 440 | 294 | 47 | 42 |
| Allergy like symptoms | 79 | 50 | 8 | 7 |
| Dyssomnias or sleep a lot | 137 | 86 | 15 | 12 |
| Weird symptoms or weakness | 581 | 358 | 62 | 51 |
| Chest congestion or pain | 289 | 178 | 31 | 25 |
| Shock | 5 | 3 | 1 | 0 |
| Sweating | 53 | 31 | 6 | 4 |
| Ear problem | 31 | 18 | 3 | 3 |
| Depression or Anxiety | 286 | 161 | 31 | 23 |
| Body aches and pain | 655 | 366 | 70 | 52 |
| Diarrhea | 65 | 36 | 7 | 5 |
| Headache or Dizziness | 375 | 205 | 40 | 29 |
| Foggy head | 53 | 28 | 6 | 4 |
| Weight loss or gain or change | 40 | 21 | 4 | 3 |
| Loss of smell and taste | 588 | 295 | 63 | 42 |
| Memory Disorders | 4 | 2 | 0 | 0 |
| Chronic obstructive lung disease | 2 | 1 | 0 | 0 |
| Cough | 432 | 216 | 46 | 31 |
| Fever | 419 | 193 | 45 | 28 |
| Nausea or vomiting | 58 | 26 | 6 | 4 |
| Cold like symptoms | 65 | 28 | 7 | 4 |
| Sputum or mucus | 90 | 38 | 10 | 5 |
| Dry throat or Sore throat | 288 | 121 | 31 | 17 |
| Stuffy or Runny nose | 230 | 92 | 25 | 13 |
| No symptoms | 16 | 6 | 2 | 1 |
| Chills | 96 | 35 | 10 | 5 |
| Menstrual problem | 4 | 1 | 0 | 0 |
| Sneezing | 36 | 8 | 4 | 1 |
| Obesity | 9 | 2 | 1 | 0 |
| Constipation | 7 | 0 | 1 | 0 |
| Epilepsy | 2 | 0 | 0 | 0 |
| Spasm | 1 | 0 | 0 | 0 |
| Anemia | 1 | 0 | 0 | 0 |

**Supplementary Table 3.** Occurrence of symptom pairs before and after COVID-19 recovery.

| **Symptom pairs** | **Occurrence (Before)** | **Occurrence (After)** | **FC (Before / After)** |
| --- | --- | --- | --- |
| Loss of smell and taste_Chills | 14.8 | 5.9 | 2.51 |
| Cough_Chills | 17.6 | 7.2 | 2.44 |
| Stuffy or Runny nose_Dry throat or Sore throat | 21.9 | 9 | 2.43 |
| Fever_Chills | 21.5 | 9 | 2.39 |
| Chills_Chest tightness or Dyspnea | 17.2 | 7.2 | 2.39 |
| Cold like symptoms_Chest tightness or Dyspnea | 12.5 | 5.4 | 2.31 |
| Chills_Body aches and pain | 21.9 | 9.5 | 2.31 |
| Dry throat or Sore throat_Chest tightness or Dyspnea | 26.2 | 11.7 | 2.24 |
| Weird symptoms or weakness_Chills | 20.7 | 9.5 | 2.18 |
| Cold like symptoms_Body aches and pain | 13.7 | 6.3 | 2.17 |
| Loss of smell and taste_Cold like symptoms | 13.7 | 6.3 | 2.17 |
| Stuffy or Runny nose_Fever | 27 | 12.6 | 2.14 |
| Headache or Dizziness_Chills | 18 | 8.6 | 2.09 |
| Depression or Anxiety_Chills | 11.3 | 5.4 | 2.09 |
| Depression or Anxiety_Cold like symptoms | 11.3 | 5.4 | 2.09 |
| Stuffy or Runny nose_Loss of smell and taste | 21.5 | 10.4 | 2.07 |
| Fever_Dry throat or Sore throat | 33.6 | 16.7 | 2.01 |
| Stuffy or Runny nose_Depression or Anxiety | 19.1 | 9.5 | 2.01 |
| Stuffy or Runny nose_Headache or Dizziness | 21.5 | 10.8 | 1.99 |
| Loss of smell and taste_Gastrointestinal symptoms | 11.7 | 5.9 | 1.98 |
| Fever_Cold like symptoms | 15.2 | 7.7 | 1.97 |
| Stuffy or Runny nose_Chest tightness or Dyspnea | 21.1 | 10.8 | 1.95 |
| Fever_Depression or Anxiety | 31.6 | 16.2 | 1.95 |
| Loss of smell and taste_Diarrhea | 10.5 | 5.4 | 1.94 |
| Stuffy or Runny nose_Allergy like symptoms | 10.5 | 5.4 | 1.94 |
| Stuffy or Runny nose_Cough | 25.4 | 13.1 | 1.94 |
| Myalgia or Fatigue_Chills | 13.7 | 7.2 | 1.90 |
| Stuffy or Runny nose_Body aches and pain | 25.4 | 13.5 | 1.88 |
| Weird symptoms or weakness_Cold like symptoms | 15.2 | 8.1 | 1.88 |
| Stuffy or Runny nose_Chest congestion or pain | 21.9 | 11.7 | 1.87 |
| Chills_Chest congestion or pain | 10.9 | 5.9 | 1.85 |
| Pneumonia_Fever | 12.5 | 6.8 | 1.84 |
| Respiratory symptoms or disease_Dry throat or Sore throat | 9.8 | 5.4 | 1.81 |
| Weird symptoms or weakness_Weight loss or gain or change | 9.8 | 5.4 | 1.81 |
| Headache or Dizziness_Dry throat or Sore throat | 27 | 14.9 | 1.81 |
| Loss of smell and taste_Fever | 37.5 | 20.7 | 1.81 |
| Dry throat or Sore throat_Depression or Anxiety | 21.9 | 12.2 | 1.80 |
| Cough_Cold like symptoms | 14.5 | 8.1 | 1.79 |
| Pneumonia_Cough | 12.1 | 6.8 | 1.78 |
| Depression or Anxiety_Cough | 30.1 | 17.1 | 1.76 |
| Skin problem_Dry throat or Sore throat | 12.5 | 7.2 | 1.74 |
| Dry throat or Sore throat_Cold like symptoms | 10.2 | 5.9 | 1.73 |
| Weird symptoms or weakness_Stuffy or Runny nose | 27.7 | 16.2 | 1.71 |
| Dry throat or Sore throat_Body aches and pain | 33.6 | 19.8 | 1.70 |
| Fever_Allergy like symptoms | 12.1 | 7.2 | 1.68 |
| Weird symptoms or weakness_Dry throat or Sore throat | 34 | 20.3 | 1.67 |
| Myalgia or Fatigue_Dry throat or Sore throat | 21.1 | 12.6 | 1.67 |
| Stuffy or Runny nose_Dyssomnias or sleep a lot | 9 | 5.4 | 1.67 |
| Weight loss or gain or change_Body aches and pain | 9 | 5.4 | 1.67 |
| Loss of smell and taste_Allergy like symptoms | 9.8 | 5.9 | 1.66 |
| Loss of smell and taste_Body aches and pain | 35.2 | 21.2 | 1.66 |
| Dry throat or Sore throat_Cough | 29.7 | 18 | 1.65 |
| Fever_Chest tightness or Dyspnea | 43.8 | 26.6 | 1.65 |
| Loss of smell and taste_Dry throat or Sore throat | 23 | 14 | 1.64 |
| Dyssomnias or sleep a lot_Dry throat or Sore throat | 12.5 | 7.7 | 1.62 |
| Fever_Body aches and pain | 48.8 | 30.2 | 1.62 |
| Weird symptoms or weakness_Fever | 51.6 | 32 | 1.61 |
| Skin problem_Cough | 14.5 | 9 | 1.61 |
| Diarrhea_Depression or Anxiety | 8.6 | 5.4 | 1.59 |
| Fever_Cough | 50 | 31.5 | 1.59 |
| Loss of smell and taste_Headache or Dizziness | 28.5 | 18 | 1.58 |
| Cough_Chest tightness or Dyspnea | 39.8 | 25.2 | 1.58 |
| Headache or Dizziness_Cough | 35.5 | 22.5 | 1.58 |
| Headache or Dizziness_Fever | 38.3 | 24.3 | 1.58 |
| Headache or Dizziness_Cold like symptoms | 12.1 | 7.7 | 1.57 |
| Loss of smell and taste_Depression or Anxiety | 23.4 | 14.9 | 1.57 |
| Depression or Anxiety_Chest tightness or Dyspnea | 26.2 | 16.7 | 1.57 |
| Stuffy or Runny nose_Gastrointestinal symptoms | 9.8 | 6.3 | 1.56 |
| Fever_Dyssomnias or sleep a lot | 19.5 | 12.6 | 1.55 |
| Dyssomnias or sleep a lot_Depression or Anxiety | 13.3 | 8.6 | 1.55 |
| Diarrhea_Body aches and pain | 12.5 | 8.1 | 1.54 |
| Depression or Anxiety_Allergy like symptoms | 9 | 5.9 | 1.53 |
| Nausea or Vomiting_Fever | 11.7 | 7.7 | 1.52 |
| Weird symptoms or weakness_Sputum or mucus | 8.2 | 5.4 | 1.52 |
| Headache or Dizziness_Diarrhea | 10.9 | 7.2 | 1.51 |
| Nausea or Vomiting_Cough | 10.9 | 7.2 | 1.51 |
| Weird symptoms or weakness_Depression or Anxiety | 34.8 | 23 | 1.51 |
| Fever_Chest congestion or pain | 35.2 | 23.4 | 1.50 |
| Loss of smell and taste_Cough | 33.2 | 22.1 | 1.50 |
| Skin problem_Loss of smell and taste | 10.2 | 6.8 | 1.50 |
| Depression or Anxiety_Body aches and pain | 30.1 | 20.3 | 1.48 |
| Gastrointestinal symptoms_Cough | 13.3 | 9 | 1.48 |
| Cough_Body aches and pain | 43.8 | 29.7 | 1.47 |
| Cold like symptoms_Chest congestion or pain | 11.3 | 7.7 | 1.47 |
| Dry throat or Sore throat_Allergy like symptoms | 11.3 | 7.7 | 1.47 |
| Loss of smell and taste_Chest tightness or Dyspnea | 27.7 | 18.9 | 1.47 |
| Mental discomfort or distress_Loss of smell and taste | 15.2 | 10.4 | 1.46 |
| Gastrointestinal symptoms_Fever | 15.2 | 10.4 | 1.46 |
| Gastrointestinal symptoms_Depression or Anxiety | 8.6 | 5.9 | 1.46 |
| Weird symptoms or weakness_Cough | 45.3 | 31.1 | 1.46 |
| Pneumonia_Body aches and pain | 12.5 | 8.6 | 1.45 |
| Pneumonia_Chest congestion or pain | 7.8 | 5.4 | 1.44 |
| Nausea or Vomiting_Headache or Dizziness | 9.8 | 6.8 | 1.44 |
| Fever_Confusion or Fluster | 9 | 6.3 | 1.43 |
| Dyssomnias or sleep a lot_Chest tightness or Dyspnea | 18 | 12.6 | 1.43 |
| Cough_Chest congestion or pain | 34 | 23.9 | 1.42 |
| Weird symptoms or weakness_Headache or Dizziness | 39.1 | 27.9 | 1.40 |
| Dry throat or Sore throat_Chest congestion or pain | 23.4 | 16.7 | 1.40 |
| Weird symptoms or weakness_Nausea or Vomiting | 11.3 | 8.1 | 1.40 |
| Weird symptoms or weakness_Loss of smell and taste | 36.7 | 26.6 | 1.38 |
| Weird symptoms or weakness_Chest tightness or Dyspnea | 42.2 | 30.6 | 1.38 |
| Headache or Dizziness_Body aches and pain | 37.9 | 27.5 | 1.38 |
| Sputum or mucus_Chest congestion or pain | 7.4 | 5.4 | 1.37 |
| Dyssomnias or sleep a lot_Cough | 17.2 | 12.6 | 1.37 |
| Chest tightness or Dyspnea_Allergy like symptoms | 9.8 | 7.2 | 1.36 |
| Nausea or Vomiting_Chest tightness or Dyspnea | 9.8 | 7.2 | 1.36 |
| Weird symptoms or weakness_Skin problem | 14.1 | 10.4 | 1.36 |
| Chest tightness or Dyspnea_Chest congestion or pain | 31.6 | 23.4 | 1.35 |
| Skin problem_Fever | 15.2 | 11.3 | 1.35 |
| Cough_Allergy like symptoms | 12.1 | 9 | 1.34 |
| Weird symptoms or weakness_Dyssomnias or sleep a lot | 21.1 | 15.8 | 1.34 |
| Chest tightness or Dyspnea_Body aches and pain | 40.2 | 30.2 | 1.33 |
| Mental discomfort or distress_Fever | 19.1 | 14.4 | 1.33 |
| Confusion or Fluster_Chest tightness or Dyspnea | 7.8 | 5.9 | 1.32 |
| Skin problem_Chest tightness or Dyspnea | 12.5 | 9.5 | 1.32 |
| Loss of smell and taste_Chest congestion or pain | 25.4 | 19.4 | 1.31 |
| Depression or Anxiety_Chest congestion or pain | 24.2 | 18.5 | 1.31 |
| Diarrhea_Chest congestion or pain | 9.4 | 7.2 | 1.31 |
| Gastrointestinal symptoms_Dry throat or Sore throat | 9.4 | 7.2 | 1.31 |
| Nausea or Vomiting_Depression or Anxiety | 9.4 | 7.2 | 1.31 |
| Skin problem_Depression or Anxiety | 9.4 | 7.2 | 1.31 |
| Mental discomfort or distress_Chills | 8.2 | 6.3 | 1.30 |
| Myalgia or Fatigue_Diarrhea | 8.2 | 6.3 | 1.30 |
| Weird symptoms or weakness_Allergy like symptoms | 11.7 | 9 | 1.30 |
| Headache or Dizziness_Dyssomnias or sleep a lot | 14.5 | 11.3 | 1.28 |
| Stuffy or Runny nose_Myalgia or Fatigue | 16.8 | 13.1 | 1.28 |
| Mental discomfort or distress_Dyssomnias or sleep a lot | 9.8 | 7.7 | 1.27 |
| Myalgia or Fatigue_Allergy like symptoms | 8.6 | 6.8 | 1.26 |
| Nausea or Vomiting_Loss of smell and taste | 8.6 | 6.8 | 1.26 |
| Sweating_Fever | 8.6 | 6.8 | 1.26 |
| Weird symptoms or weakness_Chest congestion or pain | 35.9 | 28.4 | 1.26 |
| Fever_Diarrhea | 12.5 | 9.9 | 1.26 |
| Headache or Dizziness_Gastrointestinal symptoms | 12.5 | 9.9 | 1.26 |
| Pneumonia_Chest tightness or Dyspnea | 10.2 | 8.1 | 1.26 |
| Weird symptoms or weakness_Body aches and pain | 47.3 | 37.8 | 1.25 |
| Skin problem_Body aches and pain | 14.5 | 11.7 | 1.24 |
| Headache or Dizziness_Depression or Anxiety | 22.3 | 18 | 1.24 |
| Myalgia or Fatigue_Loss of smell and taste | 22.3 | 18 | 1.24 |
| Headache or Dizziness_Chest tightness or Dyspnea | 30.1 | 24.3 | 1.24 |
| Myalgia or Fatigue_Fever | 31.2 | 25.2 | 1.24 |
| Body aches and pain_Allergy like symptoms | 11.7 | 9.5 | 1.23 |
| Weird symptoms or weakness_Diarrhea | 11.7 | 9.5 | 1.23 |
| Weird symptoms or weakness_Pneumonia | 11.7 | 9.5 | 1.23 |
| Headache or Dizziness_Chest congestion or pain | 27.7 | 22.5 | 1.23 |
| Weird symptoms or weakness_Gastrointestinal symptoms | 14.8 | 12.2 | 1.21 |
| Nausea or Vomiting_Body aches and pain | 10.9 | 9 | 1.21 |
| Sputum or mucus_Cough | 9.8 | 8.1 | 1.21 |
| Pneumonia_Headache or Dizziness | 8.2 | 6.8 | 1.21 |
| Dyssomnias or sleep a lot_Body aches and pain | 18.4 | 15.3 | 1.20 |
| Mental discomfort or distress_Dry throat or Sore throat | 12.5 | 10.4 | 1.20 |
| Myalgia or Fatigue_Headache or Dizziness | 25.8 | 21.6 | 1.19 |
| Myalgia or Fatigue_Cough | 28.5 | 23.9 | 1.19 |
| Dry throat or Sore throat_Diarrhea | 7 | 5.9 | 1.19 |
| Sputum or mucus_Body aches and pain | 7 | 5.9 | 1.19 |
| Sweating_Body aches and pain | 7 | 5.9 | 1.19 |
| Chest congestion or pain_Body aches and pain | 33.6 | 28.4 | 1.18 |
| Gastrointestinal symptoms_Chest tightness or Dyspnea | 13.3 | 11.3 | 1.18 |
| Sputum or mucus_Chest tightness or Dyspnea | 7.4 | 6.3 | 1.17 |
| Myalgia or Fatigue_Cold like symptoms | 9 | 7.7 | 1.17 |
| Chest congestion or pain_Allergy like symptoms | 9.4 | 8.1 | 1.16 |
| Dry throat or Sore throat_Anorexia | 6.2 | 5.4 | 1.15 |
| Sputum or mucus_Fever | 7.8 | 6.8 | 1.15 |
| Mental discomfort or distress_Cough | 18 | 15.8 | 1.14 |
| Headache or Dizziness_Allergy like symptoms | 8.2 | 7.2 | 1.14 |
| Gastrointestinal symptoms_Body aches and pain | 14.1 | 12.6 | 1.12 |
| Confusion or Fluster_Chest congestion or pain | 6.6 | 5.9 | 1.12 |
| Sweating_Chest tightness or Dyspnea | 6.6 | 5.9 | 1.12 |
| Diarrhea_Cough | 10.9 | 9.9 | 1.10 |
| Weird symptoms or weakness_Myalgia or Fatigue | 33.6 | 30.6 | 1.10 |
| Myalgia or Fatigue_Body aches and pain | 30.5 | 27.9 | 1.09 |
| Myalgia or Fatigue_Gastrointestinal symptoms | 9.4 | 8.6 | 1.09 |
| Dry throat or Sore throat_Autoimmune Diseases or Immunodeficiency | 5.9 | 5.4 | 1.09 |
| Dry throat or Sore throat_Confusion or Fluster | 5.9 | 5.4 | 1.09 |
| Stuffy or Runny nose_Anorexia | 5.9 | 5.4 | 1.09 |
| Sweating_Headache or Dizziness | 5.9 | 5.4 | 1.09 |
| Respiratory symptoms or disease_Fever | 13.3 | 12.2 | 1.09 |
| Skin problem_Chest congestion or pain | 9.8 | 9 | 1.09 |
| Mental discomfort or distress_Depression or Anxiety | 16.4 | 15.3 | 1.07 |
| Mental discomfort or distress_Headache or Dizziness | 16.8 | 15.8 | 1.06 |
| Skin problem_Headache or Dizziness | 10.5 | 9.9 | 1.06 |
| Myalgia or Fatigue_Chest congestion or pain | 23.8 | 22.5 | 1.06 |
| Skin problem_Mental discomfort or distress | 6.2 | 5.9 | 1.05 |
| Gastrointestinal symptoms_Diarrhea | 6.2 | 5.9 | 1.05 |
| Loss of smell and taste_Dyssomnias or sleep a lot | 10.9 | 10.4 | 1.05 |
| Diarrhea_Chest tightness or Dyspnea | 9 | 8.6 | 1.05 |
| Weird symptoms or weakness_Respiratory symptoms or disease | 14.1 | 13.5 | 1.04 |
| Myalgia or Fatigue_Chest tightness or Dyspnea | 26.6 | 25.7 | 1.04 |
| Dyssomnias or sleep a lot_Chest congestion or pain | 12.1 | 11.7 | 1.03 |
| Myalgia or Fatigue_Depression or Anxiety | 19.1 | 18.5 | 1.03 |
| Skin problem_Myalgia or Fatigue | 9.8 | 9.5 | 1.03 |
| Pneumonia_Myalgia or Fatigue | 7 | 6.8 | 1.03 |
| Weird symptoms or weakness_Sweating | 7 | 6.8 | 1.03 |
| Dry-related symptoms_Chest tightness or Dyspnea | 5.5 | 5.4 | 1.02 |
| Depression or Anxiety_Anorexia | 7.8 | 7.7 | 1.01 |
| Chest tightness or Dyspnea_Anorexia | 8.2 | 8.1 | 1.01 |
| Stuffy or Runny nose_Mental discomfort or distress | 10.9 | 10.8 | 1.01 |
| Mental discomfort or distress_Chest tightness or Dyspnea | 18.4 | 18.5 | 0.99 |
| Cough_Anorexia | 9.4 | 9.5 | 0.99 |
| Mental discomfort or distress_Anorexia | 6.2 | 6.3 | 0.98 |
| Respiratory symptoms or disease_Dyssomnias or sleep a lot | 6.2 | 6.3 | 0.98 |
| Weird symptoms or weakness_Confusion or Fluster | 7.8 | 8.1 | 0.96 |
| Confusion or Fluster_Body aches and pain | 7.4 | 7.7 | 0.96 |
| Nausea or Vomiting_Chest congestion or pain | 7.4 | 7.7 | 0.96 |
| Weird symptoms or weakness_Mental discomfort or distress | 21.1 | 22.1 | 0.95 |
| Mental discomfort or distress_Body aches and pain | 20.3 | 21.6 | 0.94 |
| Headache or Dizziness_Confusion or Fluster | 5.5 | 5.9 | 0.93 |
| Body aches and pain_Asthma | 5.5 | 5.9 | 0.93 |
| Weird symptoms or weakness_Asthma | 5.5 | 5.9 | 0.93 |
| Loss of smell and taste_Anorexia | 6.6 | 7.2 | 0.92 |
| Nausea or Vomiting_Myalgia or Fatigue | 7 | 7.7 | 0.91 |
| Fever_Anorexia | 9.8 | 10.8 | 0.91 |
| Weird symptoms or weakness_Anorexia | 9.8 | 10.8 | 0.91 |
| Myalgia or Fatigue_Dyssomnias or sleep a lot | 10.9 | 12.2 | 0.89 |
| Body aches and pain_Anorexia | 9 | 10.4 | 0.87 |
| Respiratory symptoms or disease_Cough | 10.9 | 12.6 | 0.87 |
| Others_Depression or Anxiety | 5.1 | 5.9 | 0.86 |
| Weird symptoms or weakness_Dry-related symptoms | 6.2 | 7.2 | 0.86 |
| Cough_Confusion or Fluster | 7.4 | 8.6 | 0.86 |
| Respiratory symptoms or disease_Depression or Anxiety | 7.4 | 8.6 | 0.86 |
| Mental discomfort or distress_Chest congestion or pain | 12.9 | 15.3 | 0.84 |
| Gastrointestinal symptoms_Chest congestion or pain | 8.6 | 10.4 | 0.83 |
| Myalgia or Fatigue_Mental discomfort or distress | 15.2 | 18.5 | 0.82 |
| Headache or Dizziness_Anorexia | 7.8 | 9.5 | 0.82 |
| Myalgia or Fatigue_Anorexia | 6.6 | 8.1 | 0.81 |
| Dry-related symptoms_Body aches and pain | 5.5 | 6.8 | 0.81 |
| Respiratory symptoms or disease_Body aches and pain | 11.3 | 14 | 0.81 |
| Chest congestion or pain_Anorexia | 6.2 | 7.7 | 0.81 |
| Others_Fever | 6.2 | 7.7 | 0.81 |
| Respiratory symptoms or disease_Loss of smell and taste | 7 | 9 | 0.78 |
| Respiratory symptoms or disease_Chest tightness or Dyspnea | 11.7 | 15.3 | 0.76 |
| Respiratory symptoms or disease_Headache or Dizziness | 8.6 | 11.3 | 0.76 |
| Respiratory symptoms or disease_Chest congestion or pain | 8.2 | 10.8 | 0.76 |
| Chest tightness or Dyspnea_Asthma | 5.1 | 6.8 | 0.75 |
| Respiratory symptoms or disease_Myalgia or Fatigue | 9.4 | 12.6 | 0.75 |
| Mental discomfort or distress_Gastrointestinal symptoms | 5.9 | 8.1 | 0.73 |
| Weird symptoms or weakness_Autoimmune Diseases or Immunodeficiency | 8.2 | 11.3 | 0.73 |
| Cough_Autoimmune Diseases or Immunodeficiency | 5.5 | 7.7 | 0.71 |
| Fever_Autoimmune Diseases or Immunodeficiency | 7 | 9.9 | 0.71 |
| Others_Cough | 5.5 | 8.1 | 0.68 |
| Chest tightness or Dyspnea_Autoimmune Diseases or Immunodeficiency | 6.6 | 9.9 | 0.67 |
| Others_Headache or Dizziness | 5.1 | 7.7 | 0.66 |
| Chest congestion or pain_Autoimmune Diseases or Immunodeficiency | 5.9 | 9 | 0.66 |
| Body aches and pain_Autoimmune Diseases or Immunodeficiency | 7 | 11.3 | 0.62 |
| Others_Chest tightness or Dyspnea | 6.2 | 10.4 | 0.60 |
| Headache or Dizziness_Autoimmune Diseases or Immunodeficiency | 5.1 | 8.6 | 0.59 |
| Weird symptoms or weakness_Others | 6.6 | 11.7 | 0.56 |
| Others_Body aches and pain | 5.5 | 11.3 | 0.49 |
| Mental discomfort or distress_Autoimmune Diseases or Immunodeficiency | 0 | 5.9 | 0.00 |
| Myalgia or Fatigue_Autoimmune Diseases or Immunodeficiency | 0 | 10.4 | 0.00 |
| Dyssomnias or sleep a lot_Autoimmune Diseases or Immunodeficiency | 0 | 5.9 | 0.00 |
| Loss of smell and taste_Autoimmune Diseases or Immunodeficiency | 0 | 5.9 | 0.00 |
| Skin problem_Autoimmune Diseases or Immunodeficiency | 0 | 5.9 | 0.00 |
| Respiratory symptoms or disease_Autoimmune Diseases or Immunodeficiency | 0 | 5.4 | 0.00 |
| Myalgia or Fatigue_Confusion or Fluster | 0 | 5.9 | 0.00 |
| Respiratory symptoms or disease_Mental discomfort or distress | 0 | 9.5 | 0.00 |
| Others_Mental discomfort or distress | 0 | 8.6 | 0.00 |
| Others_Myalgia or Fatigue | 0 | 9 | 0.00 |
| Chest congestion or pain_Cardiac disease and injury | 0 | 8.6 | 0.00 |
| Others_Chest congestion or pain | 0 | 7.2 | 0.00 |
| Others_Loss of smell and taste | 0 | 7.2 | 0.00 |
| Sweating_Myalgia or Fatigue | 0 | 7.2 | 0.00 |
| Headache or Dizziness_Dry-related symptoms | 0 | 6.8 | 0.00 |
| Weird symptoms or weakness_Cardiac disease and injury | 0 | 6.8 | 0.00 |
| Body aches and pain_Blood disorder | 0 | 6.3 | 0.00 |
| Cardiac disease and injury_Body aches and pain | 0 | 6.3 | 0.00 |
| Myalgia or Fatigue_Dry-related symptoms | 0 | 6.3 | 0.00 |
| Weird symptoms or weakness_Abdominal pain | 0 | 6.3 | 0.00 |
| Weird symptoms or weakness_Blood disorder | 0 | 6.3 | 0.00 |
| Body aches and pain_Abdominal pain | 0 | 5.9 | 0.00 |
| Respiratory symptoms or disease_Others | 0 | 5.9 | 0.00 |
| Chest congestion or pain_Blood disorder | 0 | 5.4 | 0.00 |
| Chest tightness or Dyspnea_Abdominal pain | 0 | 5.4 | 0.00 |
| Enlargement of lymph nodes or sinus_Body aches and pain | 0 | 5.4 | 0.00 |
| Fever_Asthma | 0 | 5.4 | 0.00 |
| Headache or Dizziness_Asthma | 0 | 5.4 | 0.00 |
| Myalgia or Fatigue_Blood disorder | 0 | 5.4 | 0.00 |
| Myalgia or Fatigue_Cardiac disease and injury | 0 | 5.4 | 0.00 |
| Nerve Pain and disease_Body aches and pain | 0 | 5.4 | 0.00 |
| Nerve Pain and disease_Chest tightness or Dyspnea | 0 | 5.4 | 0.00 |
| Nerve Pain and disease_Myalgia or Fatigue | 0 | 5.4 | 0.00 |
| Respiratory symptoms or disease_Gastrointestinal symptoms | 0 | 5.4 | 0.00 |
| Skin problem_Gastrointestinal symptoms | 0 | 5.4 | 0.00 |
| Sputum or mucus_Myalgia or Fatigue | 0 | 5.4 | 0.00 |
| Weird symptoms or weakness_Nerve Pain and disease | 0 | 5.4 | 0.00 |
| Depression or Anxiety_Autoimmune Diseases or Immunodeficiency | 5.1 | 0 | - |
| Mental discomfort or distress_Allergy like symptoms | 5.9 | 0 | - |
| Nausea or Vomiting_Mental discomfort or distress | 5.9 | 0 | - |
| Mental discomfort or distress_Cold like symptoms | 5.1 | 0 | - |
| Mental discomfort or distress_Diarrhea | 5.1 | 0 | - |
| Loss of smell and taste_Confusion or Fluster | 6.2 | 0 | - |
| Depression or Anxiety_Confusion or Fluster | 5.9 | 0 | - |
| Dry throat or Sore throat_Chills | 13.7 | 0 | - |
| Stuffy or Runny nose_Cold like symptoms | 10.9 | 0 | - |
| Sneezing_Cough | 9.8 | 0 | - |
| Sneezing_Fever | 9.8 | 0 | - |
| Stuffy or Runny nose_Skin problem | 9.4 | 0 | - |
| Weight loss or gain or change_Fever | 9 | 0 | - |
| Weird symptoms or weakness_Sneezing | 9 | 0 | - |
| Stuffy or Runny nose_Chills | 8.6 | 0 | - |
| Sweating_Cough | 8.6 | 0 | - |
| Weight loss or gain or change_Chest tightness or Dyspnea | 8.6 | 0 | - |
| Weight loss or gain or change_Cough | 8.6 | 0 | - |
| Dyssomnias or sleep a lot_Chills | 8.2 | 0 | - |
| Pneumonia_Depression or Anxiety | 8.2 | 0 | - |
| Sneezing_Body aches and pain | 8.2 | 0 | - |
| Sneezing_Depression or Anxiety | 7.8 | 0 | - |
| Stuffy or Runny nose_Diarrhea | 7.8 | 0 | - |
| Eye problem_Body aches and pain | 7.4 | 0 | - |
| Gastrointestinal symptoms_Dyssomnias or sleep a lot | 7.4 | 0 | - |
| Nausea or Vomiting_Chills | 7.4 | 0 | - |
| Cold like symptoms_Chills | 7 | 0 | - |
| Pneumonia_Dry throat or Sore throat | 7 | 0 | - |
| Pneumonia_Dyssomnias or sleep a lot | 7 | 0 | - |
| Sneezing_Chest tightness or Dyspnea | 7 | 0 | - |
| Sputum or mucus_Depression or Anxiety | 7 | 0 | - |
| Weird symptoms or weakness_Eye problem | 7 | 0 | - |
| Diarrhea_Chills | 6.6 | 0 | - |
| Eye problem_Cough | 6.6 | 0 | - |
| Fever_Eye problem | 6.6 | 0 | - |
| Gastrointestinal symptoms_Chills | 6.6 | 0 | - |
| Skin problem_Chills | 6.6 | 0 | - |
| Sneezing_Dry throat or Sore throat | 6.6 | 0 | - |
| Sneezing_Loss of smell and taste | 6.6 | 0 | - |
| Sweating_Dyssomnias or sleep a lot | 6.6 | 0 | - |
| Weight loss or gain or change_Headache or Dizziness | 6.6 | 0 | - |
| Nausea or Vomiting_Dry throat or Sore throat | 6.2 | 0 | - |
| Sneezing_Headache or Dizziness | 6.2 | 0 | - |
| Weight loss or gain or change_Chest congestion or pain | 6.2 | 0 | - |
| Weight loss or gain or change_Dry throat or Sore throat | 6.2 | 0 | - |
| Weight loss or gain or change_Loss of smell and taste | 6.2 | 0 | - |
| Dry-related symptoms_Cough | 5.9 | 0 | - |
| Fever_Dry-related symptoms | 5.9 | 0 | - |
| Gastrointestinal symptoms_Cold like symptoms | 5.9 | 0 | - |
| Headache or Dizziness_Eye problem | 5.9 | 0 | - |
| Nausea or Vomiting_Diarrhea | 5.9 | 0 | - |
| Pneumonia_Loss of smell and taste | 5.9 | 0 | - |
| Sputum or mucus_Headache or Dizziness | 5.9 | 0 | - |
| Stuffy or Runny nose_Nausea or Vomiting | 5.9 | 0 | - |
| Stuffy or Runny nose_Respiratory symptoms or disease | 5.9 | 0 | - |
| Stuffy or Runny nose_Sputum or mucus | 5.9 | 0 | - |
| Dry-related symptoms_Depression or Anxiety | 5.5 | 0 | - |
| Dyssomnias or sleep a lot_Diarrhea | 5.5 | 0 | - |
| Eye problem_Chest tightness or Dyspnea | 5.5 | 0 | - |
| Eye problem_Dry throat or Sore throat | 5.5 | 0 | - |
| Loss of smell and taste_Dry-related symptoms | 5.5 | 0 | - |
| Others_Dry throat or Sore throat | 5.5 | 0 | - |
| Skin problem_Allergy like symptoms | 5.5 | 0 | - |
| Sneezing_Myalgia or Fatigue | 5.5 | 0 | - |
| Sputum or mucus_Loss of smell and taste | 5.5 | 0 | - |
| Stuffy or Runny nose_Sneezing | 5.5 | 0 | - |
| Weight loss or gain or change_Depression or Anxiety | 5.5 | 0 | - |
| Weight loss or gain or change_Myalgia or Fatigue | 5.5 | 0 | - |
| Weird symptoms or weakness_Ear problem | 5.5 | 0 | - |
| Cold like symptoms_Allergy like symptoms | 5.1 | 0 | - |
| Diarrhea_Cold like symptoms | 5.1 | 0 | - |
| Dyssomnias or sleep a lot_Allergy like symptoms | 5.1 | 0 | - |
| Dyssomnias or sleep a lot_Anorexia | 5.1 | 0 | - |
| Ear problem_Body aches and pain | 5.1 | 0 | - |
| Fever_Ear problem | 5.1 | 0 | - |
| Nausea or Vomiting_Anorexia | 5.1 | 0 | - |
| Nausea or Vomiting_Dyssomnias or sleep a lot | 5.1 | 0 | - |
| No symptoms_Fever | 5.1 | 0 | - |
| Others_Dyssomnias or sleep a lot | 5.1 | 0 | - |
| Respiratory symptoms or disease_Chills | 5.1 | 0 | - |
| Sneezing_Chest congestion or pain | 5.1 | 0 | - |
| Sneezing_Cold like symptoms | 5.1 | 0 | - |
| Stuffy or Runny nose_Eye problem | 5.1 | 0 | - |
| Sweating_Depression or Anxiety | 5.1 | 0 | - |

**Supplementary Table 4.** Symptom duration before and after COVID-19 recovery.

| **symptom** | **mean_duration_before** | **mean_duration_after** |
| --- | --- | --- |
| Abdominal pain | 49 | 25 |
| Acute respiratory distress syndrome | 77 | 0 |
| Allergy like symptoms | 28 | 53 |
| Anemia | 10 | - |
| Anorexia | 24 | 25 |
| Arthritis | 23 | 17 |
| Asthma | 31 | 68 |
| Autoimmune Diseases or Immunodeficiency | 32 | 61 |
| Blood disorder | 22 | 40 |
| Blood pressure-related | 25 | 45 |
| Body aches and pain | 55 | 65 |
| Cancer | 39 | 40 |
| Cardiac disease and injury | 32 | 58 |
| Chest congestion or pain | 44 | 55 |
| Chest tightness or Dyspnea | 55 | 57 |
| Chills | 44 | 49 |
| Cold like symptoms | 36 | 62 |
| Confusion or Fluster | 18 | 62 |
| Constipation | 40 | - |
| Cough | 52 | 47 |
| Depression or Anxiety | 44 | 42 |
| Diabetes | 16 | 23 |
| Diarrhea | 26 | 42 |
| Dry throat or Sore throat | 51 | 70 |
| Dry-related symptoms | 23 | 46 |
| Dyssomnias or sleep a lot | 43 | 34 |
| Ear problem | 24 | 23 |
| Enlargement of lymph nodes or sinus | 32 | 39 |
| Epilepsy | 16 | - |
| Eye problem | 46 | 32 |
| Fever | 54 | 62 |
| Finger or Toes problem | 21 | 39 |
| Gastrointestinal symptoms | 44 | 61 |
| Headache or Dizziness | 54 | 65 |
| Loss of smell and taste | 37 | 55 |
| Lung disease | 32 | 69 |
| Menstrual problem | 12 | 13 |
| Mental discomfort or distress | 42 | 38 |
| Myalgia or Fatigue | 50 | 58 |
| Nausea or vomiting | 37 | 45 |
| Nerve Pain and disease | 23 | 37 |
| No symptoms | 28 | 52 |
| Obesity | 13 | 70 |
| Oral problem | 13 | 59 |
| Others | 49 | 30 |
| Pneumonia | 37 | 55 |
| Renal symptoms or disease | 29 | 29 |
| Respiratory symptoms or disease | 46 | 52 |
| Sepsis | 14 | 24 |
| Shock | 25 | 15 |
| Skin problem | 42 | 43 |
| Sneezing | 35 | 75 |
| Spasm | 87 | - |
| Sputum or mucus | 20 | 62 |
| Stroke | 59 | 30 |
| Stuffy or Runny nose | 48 | 48 |
| Sweating | 33 | 114 |
| Weight loss or gain or change | 27 | 20 |
| Weird symptoms or weakness | 54 | 66 |
| Wounds and Injuries | 122 | 28 |
|  |  |  |
